# designGG: an R-package and web tool for the optimal design of genetical genomics experiments

**DOI:** 10.1186/1471-2105-10-188

**Published:** 2009-06-18

**Authors:** Yang Li, Morris A Swertz, Gonzalo Vera, Jingyuan Fu, Rainer Breitling, Ritsert C Jansen

**Affiliations:** 1 Groningen Bioinformatics Center, Groningen Biomolecular Sciences and Biotechnology Institute, University of Groningen, Haren, The Netherlands; 2 Department of Genetics, University Medical Center Groningen and University of Groningen, Groningen, The Netherlands

## Abstract

**Background:**

High-dimensional biomolecular profiling of genetically different individuals in one or more environmental conditions is an increasingly popular strategy for exploring the functioning of complex biological systems. The optimal design of such genetical genomics experiments in a cost-efficient and effective way is not trivial.

**Results:**

This paper presents designGG, an R package for designing optimal genetical genomics experiments. A web implementation for designGG is available at . All software, including source code and documentation, is freely available.

**Conclusion:**

DesignGG allows users to intelligently select and allocate individuals to experimental units and conditions such as drug treatment. The user can maximize the power and resolution of detecting genetic, environmental and interaction effects in a genome-wide or local mode by giving more weight to genome regions of special interest, such as previously detected phenotypic quantitative trait loci. This will help to achieve high power and more accurate estimates of the effects of interesting factors, and thus yield a more reliable biological interpretation of data. DesignGG is applicable to linkage analysis of experimental crosses, e.g. recombinant inbred lines, as well as to association analysis of natural populations.

## Background

Genetical genomics [1] has become a popular strategy for studying complex biological systems using a combination of classical genetics, biomolecular profiling and bioinformatics [2-5]. By measuring molecular variation, using transcriptomics, proteomics, metabolomics and related emerging technologies, in genetically different individuals, genetical genomics has the potential to identify the functional consequences of natural and induced genetic variation. Recently, genetical genomics has been generalized to achieve a comprehensive understanding of the dynamics of molecular networks by combining environmental and genetic perturbation [6,7]. This type of large scale "omics" study leads to a better understanding of why individuals of the same species respond differently to drugs, pathogens, and other environmental factors.

However, most molecular profiling experiments are very costly, and as a consequence most genetical genomics studies are performed at the verge of statistical feasibility. Therefore, experimental design needs careful consideration to achieve maximum power from limited resources, such as microarrays and experimental animals [8,9]. But, even in standard scenarios this requires sophisticated application of statistical concepts to intelligently select genetically different individuals from a population and allocate them to different conditions and experimental units. This topic has motivated classical statistical research since a long time [10]. More recently, the concepts developed there have been adapted to the high dimensional data sets of post-genomics research [8,11-13], and useful simplified design strategies have been suggested [11,14]. However, to transfer these statistical ideas to the even more complex context of genetical genomics [9,15,16] still requires considerable expertise in statistics.

Here we present an online web tool to make these selections and allocations easy for biologists with little/no statistical training. The program will find the best experimental design to produce the most accurate estimates of the most relevant biological parameters, given the number of experimental factors to be varied, the genotype information on the population, the profiling technology used, and the constraints on the number of individuals that can be profiled. Advanced users can download the underlying methods as an R package to adapt the program for a more tailored design. Without loss of generality, we will illustrate the method using microarrays, while they apply equally well to other profiling technologies, such as mass spectrometry. Also, we will only discuss molecular technologies that profile samples individually (e.g., single color microarrays) or in pairs (e.g., dual color microarrays), but an extension of the R scripts to more advanced multiplex technologies would be straightforward [17].

## Implementation

The objective of designGG is to find an optimal allocation of genetically different samples to different conditions and experimental units (arrays) favoring a precise estimate of interesting parameters, such as main genetic effects and interaction effects between genotype and drug treatment. A simple case with one environmental factor can be expressed as y = μ + G× E + ε, where y is the measurement vector, ε is the error term, and G×E denotes main effect and interaction effects of genotype and environment. In matrix notation, a model with one or more genotype factors (quantitative trait loci; QTL) and one or more environmental factors can be written as: **Y **= **Xβ **+ **E**, where **X **is the design matrix of samples by parameters and **β **is the effect of genotype and environmental factors. The least squares estimate of **β **is b = (**X**^T^**X**)^-1^**X**^T^**Y **with var(b) = σ^2^(**X**^T^**X**)^-1^. The optimal experiment design is defined as the one that minimizes the double sum of the variances of b firstly summed over all parameters and then summed over all genotypic markers. We use an optimization algorithm (simulated annealing [18]) to search the experimental design space of all possible allocations to produce an optimal design matrix **X**. During the optimization, the algorithm utilizes the available marker information from the individuals to optimize the allocation of individuals to microarrays and conditions.

In the optimization, the experimenter can, of course, give more weight to parameters of higher interest, which will then be estimated with higher accuracy. Particularly, prior knowledge about expected effect sizes of interesting factors can be incorporated as weight parameters for the algorithm and the weight is inversely proportional to the expected effect size of the corresponding factors. In addition, it is also possible to specify the genome regions that are of major interest in a particular experiment, by specifying a region parameter. For example, if the relevant phenotype is known to map to certain genome regions, parameters for the markers in these regions can be given full weight in the optimization algorithm, whereas parameters for other markers can be given lesser or even zero weight. Thus, mapping resolution can improve and the power for finding QTLs in focal regions can be increased.

DesignGG is a package entirely written in the R language [19]. Every function of the designGG library is available as a stand-alone R tool and detailed help is available according to the standard format of R documentation.

## Results

### Web tool

**Figure 1:**
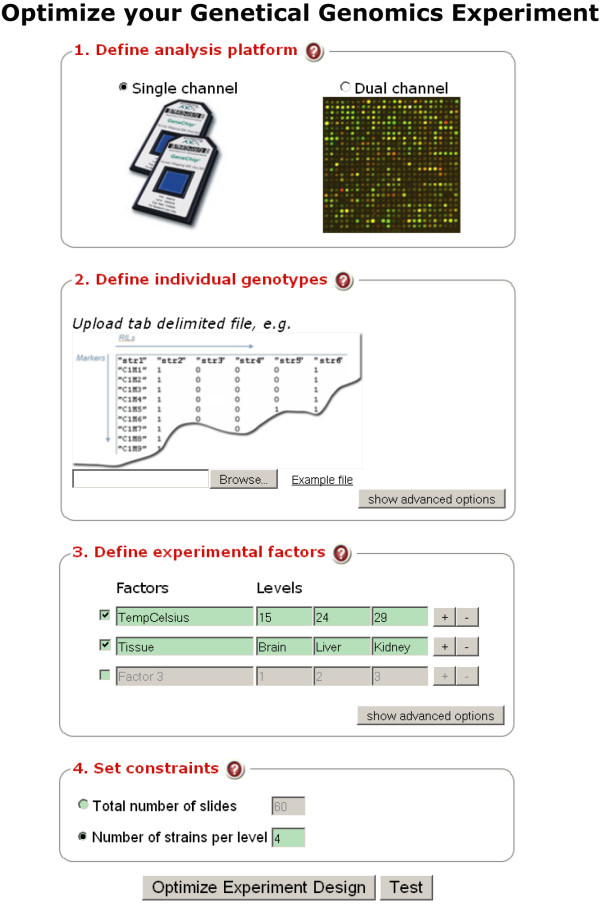
**Screenshot of the designGG web interface**.

Users can apply this method using a web interface (Figure 1) that we have generated using MOLGENIS [20,21]:

1. Choose the platform. Select the single- or dual-channel option for one-color or two-color gene expression microarrays (the dual-channel option is also used for any other technology profiling pairs of samples).

2. Upload a tab separated value (TXT) file containing the genotype data matrix (individuals × markers). Each cell contains a genotype label (e.g. A or B for the parental alleles, H for heterozygous loci; NA for missing data).

3. Set parameters. Specify the number of environmental factors, their number of levels, and the possible values of these levels. Specify either the total number of slides (assays) or the number of samples allocated within each condition.

4. Use advanced options if only one or a few genome regions or particular factors are of major interest. It is possible to optimize the experimental design by focusing on certain regions (e.g. the first 20 markers on chromosome I). Prior knowledge about expected effect sizes of interesting factors can also be incorporated as weight parameters for the algorithm.

5. Start the optimization algorithm by clicking on the button **Optimize Experimental Design **(Figure 1).

6. Get results. After the optimization is finished, the optimal experimental design will be displayed online (in table format), and will be available as text files for download.

### R package

Here we illustrate how to apply the designGG R package using an example: suppose we are studying the effect of genetic factors (Q), temperature (F_1_), drug treatment (F_2_) and their interaction on gene expression using two-colour microarrays. There are 100 microarray slides available for this experiment, and we plan to study two different levels for each environment, which are 16°C and 24°C for F_1 _(temperature), and 5 μM and 10 μM for F_2 _(drug treatment). Then the R package can also be used in command line form as follows:

1. Prepare the input file specifying the genotype of each individual at each marker position. The file should be formatted as tab separated values (TXT), as illustrated in Table 1.

**Table 1:** Example table of genotype data. Heterozygous loci are indicated by an H.

	**Strain 1**	**Strain 2**	**Strain 3**	**Strain 4**	**Strain 5**	...
C1M1	A	B	B	B	A	...

C1M2	A	H	A	B	A	...

C1M3	A	A	B	H	A	...

...	...	...	...	...	...	...

2. Load the designGG package by starting the R application and typing the command:

> library(designGG)

Specify the input arguments (Steps 3–5 correspond to steps 2–4 of using the web tool. The order of the following commands in steps 3–5 does not matter).

3. Choose the platform of the experiment. In this example, we use two-color microarray, thus:

> bTwoColorArray   <- T #if paired; F otherwise

4. Load the marker data and specify the following required arguments (number of environmental factors, number of levels per factor, the values of each level, and the number of available slides):

> data(genotype) #an example data attached with the designGG package

# The command below can be used to read TXT data

# genotype      <- read.table("genotype.txt")

> nEnvFactors   <- 2

> nLevels   <- c(2, 2)

> Level      <- list(c(16, 24), c(5, 10))

> nSlides      <- 100; nTuple <- NULL

An alternative to specifying nSlides is to specify nTuple, the number of strains to be allocated onto each condition. For example,

> nTuple      <- 25 ; nSlides <- NULL;

5. In addition to the required arguments specified in step 4, there are some optional ones for a tailored experimental design: e.g., we might be especially interested in the genome region between 1^st ^marker and 20^th ^marker, where a known phenotypic QTL from previous study locates. They can then specify that the optimization algorithm should only take genotypes at markers 1 to 20 into account:

> region      <- seq(1, 20, by = 1)

Additionally, if we want that the estimates of all interaction effects are twice as accurate as the estimates of the main effects (genotype, temperature and drug treatment), then we specify weights for the estimates:

> weight      <- c(0.5,0.5,0.5,1,1,1,1)

Here the order of elements in the weight vector is such that first the main effects are listed, starting with the genotype, followed by the two environmental factors in the order used for nLevels and Level, then the one-way interactions, in the same order, and finally the two-way interaction between all three factors.

6. The following commands specify the directory where the resulting optimal design tables are to be stored and the name of the output files (design tables):

> directory   <- "C:\myproject\design"

> fileName      <- "myDesign"

A detailed explanation of the above arguments can also be found in Table 2.

**Table 2:** The description and possible values of designGG arguments

**Arguments**	**Description**	**Possible value(s)**
bTwoColorArray^a^	The type of platform	T(RUE) or F(ALSE) for the dual- or single-channel option, respectively. For example, F for one-color and T for two-color gene expression microarrays (the dual-channel option is also used for any other technology profiling pairs of samples)

genotype^a^	Genotype information	A matrix of marker genotypes for each marker and each strain. The values can be numeric: "1" and "0" for two homozygous genotypes, respectively (optionally, "0.5" for heterozygous allele). They can also be characters: "A" "B" or "H" and "H" is for heterozygous allele; NA for missing data. The column names are strain names, such as "Strain 1", "Strain 2", etc. The row names are marker names, such as "C1M1", "C2M2", etc.

nEnvFactors^a^	Number of environmental factors in the study	A numeric integer value between 1 and 3 which indicates the number of environmental factors to be studied. Experiments with more than three environmental factor are not recommended here since the power to estimate the high-order interactions is very limited for a realistic number of samples (several hundreds).

nLevels^a^	Number of levels for each environmental factor	A numeric integer vector. For example, there are two different levels for two environmental factors under study, then we use nLevels <- c(2, 2)

Level^b^	Level values for each environmental factor	A list which specifies the levels for each factor in the experiment. The element is a vector describing all levels of the environmental factor. In the given example, temperature levels are 16 and 24 and drug treatment levels are 5 and 10. The we use: Level <- list(c(16, 24), c(5, 10))

nSlides^c^	Total number of slides available for the experiment.	A numeric integer value

nTuple^c^	Average number of strains to be assigned onto each condition	A numeric value which is larger than 1

region^b^	Genome region of biological interest	A numeric integer vector which indicates the markers of biological interest, for example those previously detected for phenotypic quantitative trait loci. The value is the marker index (i.e., the row number in the genotype data table), *not *the marker name.

weight^b^	The weights for estimating genetic and environmental factors, and their interaction terms	A numeric vector which indicates the parameters of biological interest. Higher weights correspond to higher interest, and the optimization is adjusted in such a way as to result in a higher accuracy of the estimate for the parameters with higher weight. Prior knowledge about expected effect sizes of interesting factors can also be incorporated as weight parameters for the algorithm. The weight is inversely proportional to the expected effect size of the corresponding parameter, if the same relative accuracy is intended. When there is no environmental perturbation, weights is 1, as there is only one parameter of interest (genotype); When nEnvFactor = 1, weight = c(w_Q_, w_F1_, w_QF1_); When nEnvFactor = 2, weight = c(w_Q_, w_F1_, w_F2_, w_QF1_, w_QF2_, w_F1F2_, w_QF1F2_); When nEnvFactor = 3, weight = c(w_Q_, w_F1_, w_F2_, w_F2_, w_QF1_, w_QF2_, w_QF3_, w_F1F2_, w_F1F3_, w_F2F3_, w_QF1F2_, w_QF1F3_, w_QF2F3_, w_QF1F2F3_). Here w_Q _represents the weight for genotype effect, w_F1 _represents the weight for environmental factor F_1 _effect and w_QF1 _represents the weight for interaction between genotype and F_1 _effect, etc.

nIterations^b^	Number of iterations of the simulated annealing method	A numeric integer value larger than 1. Default = 3000

directory^b^	Output file directory	The path where output files will be saved.

fileName^b^	Output file names	The name for output tables in CSV format to be produced.

^a^Required input arguments from users

^b^Optional input arguments

^c^Alternative arguments: either of them is required

7. Run designGG to obtain your optimal design:

> myOutput <- designGG(genotype, nSlides, nTuple, nEnvFactors, nLevels, Level, region = region, weight = weight, nIterations = 10)

It should be noted that the number of iteration of the simulated annealing method (fnIterations)is set to 10 here for testing purposes. The default value (nIterations = 3000) is recommended, but it will result in a longer computing time.

8. Output can be found in the directory or retrieved with:

> optimalArrayDesign   <- myOutput$arrayDesign

> optimalCondDesign   <- myOutput$conditionDesign

Example output tables for allocation of strains on arrays and different conditions are shown in Table 3 and 4, respectively.

**Table 3:** Example table of the allocation of strains to arrays.

	**Channel 1**	**Channel 2**
array 1	Strain 28	Strain 92

array 2	Strain 70	Strain 47

array 3	Strain 22	Strain 89

...	...	...

This is applicable for technologies that profile samples in pairs, e.g. two-color microarrays.

**Table 4:** Example table of the allocation of strains to experimental conditions.

	Temperature	Drug	Selected Strains				
condition 1	16	5	Strain 28	Strain 81	Strain 18	Strain 61	...

condition 2	24	5	Strain 70	Strain 40	Strain 83	Strain 92	...

condition 3	24	10	Strain 14	Strain 3	Strain 89	Strain 22	...

...	...	...	...	...	...	...	...

If the number of strains is smaller than the number of combinations of factors, the same strain can be used multiple times.

9. In addition, users can check the curve of optimization score recorded as the algorithm iterates using:

> plotAllScores (myOutput$plot.obj)

Details of default settings such as method (SA: simulated annealing) or nSearch (equals 2) can be found in the designGG manual or the online help. Example genotype data and output tables are also provided along with the package. The R package can be found in Additional file 1 and most up-to-date version of the software can be downloaded at .

### Expected Results

Two tables summarize the optimal design: The table pair design is only used for two-channel experiments and describes how samples are paired together in one assay e.g., a two-color microarray chip (Table 3). The table environment design lists how samples are assigned to environments/experimental factors (Table 4).

## Conclusion

DesignGG, a freely-available R package and web tool presented in this work, represents a novel tool for the researcher interested in system genetics. Based on the careful experimental design provided by designGG, limited resources, such as arrays and samples, are maximally exploited, and more accurate estimates of parameters of interest can be achieved.

## Availability and requiredments

Project name: designGG R package and web tool

Project home page: 

Programming language: R

Requirement: R statistical software available at  for the stand-alone version.

## Authors' contributions

YL developed designGG. RCJ and RB directed the project. MAS, GV and JF helped to implement the web tool. All authors wrote the manuscript, and read and approved the final version.

## Supplementary Material

Additional file 1 **designGG: an R-package for the optimal design of genetical genomics experiments**. DesignGG aims at finding an optimal design of genetical genomics experiments which maximize the power and resolution of detecting genetic, environmental and interaction effects. This will help to achieve high power and more accurate estimates of the effects of interesting factors, and thus yield a more reliable biological interpretation of data. Click here for file
