# Supplementary material for: designGG: an R-package and web tool for the optimal design of genetical genomics experiments
Source: BMC Bioinformatics. 2009 Jun 18;10:188. doi: 10.1186/1471-2105-10-188 (PMC2706229; doi:10.1186/1471-2105-10-188)
Supplement: Additional file 1 — designGG: an R-package for the optimal design of genetical genomics experiments. DesignGG aims at finding an optimal design of genetical genomics experiments which maximize the power and resolution of detecting genetic, environmental and interaction effects. This will help to achieve high power and more accurate estimates of the effects of interesting factors, and thus yield a more reliable biological interpretation of data. [file 1471-2105-10-188-S1.zip › designGG/html/00Index.html]

R: Computational tool for designing genetical genomics experiments.

# Computational tool for designing genetical genomics experiments.

---

## Documentation for package ‘designGG’ version 1.0-02

## Help Pages

|  |  |
| --- | --- |
| acceptanceProbability | Compute the acceptance probability for each updated design |
| arrayUpdate | Update array allocation |
| conditionAllocation | Allocate the selected RILs into different conditions |
| conditionCombination | Generate a matrix indicating all possible levels for environmental factors |
| conditionLevel | Levels of all environmental factors |
| conditionUpdate | Update condition allocation |
| design genetical genomics experiment | Optimal design for genetical genomics experiments |
| designGG | Optimal design for genetical genomics experiments |
| designScore | Calculate the A- or D- optimality score based on current experimental design |
| exampleArrayDesignTable | Example output of ArrayDesignTable data |
| exampleConditionDesignTable | Example ConditionDesignTable data |
| examplePlotObj | Example PlotObj data |
| experimentDesignTable | Make experiment table based design matrix |
| genotype | Example genotype data |
| initialDesign | Initialize an experiment design matrix |
| interactionLevel | Generate levels for all interacting factors |
| pairLevel | Pair levels for paired RILs (or strains) |
| plotAllScores | Plot scores profiles |
| temperatureStep | Calculate the temperature decreasing step for simulated annealing process |
| updateDesign | Updates current design |
| variableNames | Generate variable names for all factors |
| variableNumber | Compute the number of variables in the experiment |
