# Supplementary material for: designGG: an R-package and web tool for the optimal design of genetical genomics experiments
Source: BMC Bioinformatics. 2009 Jun 18;10:188. doi: 10.1186/1471-2105-10-188 (PMC2706229; doi:10.1186/1471-2105-10-188)
Supplement: Additional file 1 — designGG: an R-package for the optimal design of genetical genomics experiments. DesignGG aims at finding an optimal design of genetical genomics experiments which maximize the power and resolution of detecting genetic, environmental and interaction effects. This will help to achieve high power and more accurate estimates of the effects of interesting factors, and thus yield a more reliable biological interpretation of data. [file 1471-2105-10-188-S1.zip › designGG/html/acceptanceProbability.html]

R: Compute the acceptance probability for each updated design

|  |  |
| --- | --- |
| acceptanceProbability {designGG} | R Documentation |

## Compute the acceptance probability for each updated design

### Description

Compute the acceptance probability for each updated design. It depends on the
current temperature value of simulated annealing process.
This is a subfunction needed for `designGG`, but is not directly used.

### Usage

```
    acceptanceProbability( designScore, newDesignScore, method, 
                           temperature )
```

### Arguments

|  |  |
| --- | --- |
| `designScore` | score of current design. |
| `newDesignScore` | score of updated design. |
| `method` | either "SA" (simulated annealing) or "MH". (Metropolis Hastings) |
| `temperature` | current temperature in simulated annealing process. |

### Author(s)

Yang Li <yang.li@rug.nl>, Gonzalo Vera <gonzalo.vera.rodriguez@gmail.com>   
Rainer Breitling <r.breitling@rug.nl>, Ritsert Jansen <r.c.jansen@rug.nl>

### References

E. Wit and J. McClure. Statistics for Microarrays: Design, Analysis
and Inference. (2004) Chichester: Wiley.   
Y. Li, R. Breitling and R.C. Jansen. Generalizing genetical
genomics: the added value from environmental perturbation, Trends Genet
(2008) 24:518-524.   
Y. Li, M. Swertz, G. Vera, J. Fu, R. Breitling, and R.C. Jansen. designGG:
An R-package and Web tool for the optimal design of genetical genomics
experiments. (submitted)   
http://gbic.biol.rug.nl/designGG

### See Also

`designGG`

---

[Package *designGG* version 1.0-02 Index]
