# Supplementary material for: designGG: an R-package and web tool for the optimal design of genetical genomics experiments
Source: BMC Bioinformatics. 2009 Jun 18;10:188. doi: 10.1186/1471-2105-10-188 (PMC2706229; doi:10.1186/1471-2105-10-188)
Supplement: Additional file 1 — designGG: an R-package for the optimal design of genetical genomics experiments. DesignGG aims at finding an optimal design of genetical genomics experiments which maximize the power and resolution of detecting genetic, environmental and interaction effects. This will help to achieve high power and more accurate estimates of the effects of interesting factors, and thus yield a more reliable biological interpretation of data. [file 1471-2105-10-188-S1.zip › designGG/html/arrayUpdate.html]

R: Update array allocation

|  |  |
| --- | --- |
| arrayUpdate {designGG} | R Documentation |

## Update array allocation

### Description

Update the allocation of samples on the arrays.
This is a subfunction needed for `updateDesign`, but is not directly used.

### Usage

```
  arrayUpdate(array.allocation, condition.allocation, nRILs, nSlides)
```

### Arguments

|  |  |
| --- | --- |
| `array.allocation` | matrix with nArray rows and nRIL columns. Elements of 1/0 indicate this RIL (or strain) is/not selected for this array. |
| `condition.allocation` | matrix with nCondition rows and nRIL columns. Elements of 1/0 indicate this RIL (or strain) is/not selected for this condition. |
| `nRILs` | number of RILs or strains available for the experiment. |
| `nSlides` | total number of slides available for experiment. |

### Details

This function is used only for designing a dual-channel experiment where samples
need to be paired.

### Value

A list with the following two elements:   
`new.array.allocation`: an updated array allocation table   
`new.condition.allocation`: an updated condition allocation table

### Author(s)

Yang Li <yang.li@rug.nl>, Gonzalo Vera <gonzalo.vera.rodriguez@gmail.com>   
Rainer Breitling <r.breitling@rug.nl>, Ritsert Jansen <r.c.jansen@rug.nl>

### References

Y. Li, R. Breitling and R.C. Jansen. Generalizing genetical
genomics: the added value from environmental perturbation, Trends Genet
(2008) 24:518-524.   
Y. Li, M. Swertz, G. Vera, J. Fu, R. Breitling, and R.C. Jansen. designGG:
An R-package and Web tool for the optimal design of genetical genomics
experiments. (submitted)   
http://gbic.biol.rug.nl/designGG

### See Also

`updateDesign`

---

[Package *designGG* version 1.0-02 Index]
