# Supplementary material for: designGG: an R-package and web tool for the optimal design of genetical genomics experiments
Source: BMC Bioinformatics. 2009 Jun 18;10:188. doi: 10.1186/1471-2105-10-188 (PMC2706229; doi:10.1186/1471-2105-10-188)
Supplement: Additional file 1 — designGG: an R-package for the optimal design of genetical genomics experiments. DesignGG aims at finding an optimal design of genetical genomics experiments which maximize the power and resolution of detecting genetic, environmental and interaction effects. This will help to achieve high power and more accurate estimates of the effects of interesting factors, and thus yield a more reliable biological interpretation of data. [file 1471-2105-10-188-S1.zip › designGG/html/conditionAllocation.html]

R: Allocate the selected RILs into different conditions

|  |  |
| --- | --- |
| conditionAllocation {designGG} | R Documentation |

## Allocate the selected RILs into different conditions

### Description

This is a subfunction used by `initialDesign` but is not directly used.
In the experiment where samples are profiled in pairs, the samples are firstly
selected and paired on each array and then the selected samples are randomly allocated
into different conditions.

### Usage

```
  conditionAllocation( selectedRILs, genotype, nConditions, nSlides, nTuple )
```

### Arguments

|  |  |
| --- | --- |
| `selectedRILs` | the index of the selected RILs or strains among all that are available for the experiment. |
| `genotype` | genotype data: a nMarker-by-nRILs matrix with two allels being 0 and 1 (or A and B) or three allels being 0, 0.5 and 1 (or, A, H, and B), where 0.5 (or H) represents heterozygous allele. |
| `nConditions` | number of all possible combination of all environmental factors. It should be larger than 1. |
| `nSlides` | total number of slides available for the experiment. It should be a non-zero integer. |
| `nTuple` | average number of RILs to be assigned onto each condition.   `nTuple` should be a real number which is larger than 1.  if `nTuple` < 1, the algorithm will stop and show a message as below,   `warning: "The number of slides is too small to perform the experiment."` |

### Details

This function is only called by `initialDesign` function when `btwoColorArray`
is `TRUE`.

### Value

A matrix with nCondition rows and nRIL columns.
Elements of 1/0 indicate that this RIL (or strain) is/not selected for this condition.

### Author(s)

Yang Li <yang.li@rug.nl>, Gonzalo Vera <gonzalo.vera.rodriguez@gmail.com>   
Rainer Breitling <r.breitling@rug.nl>, Ritsert Jansen <r.c.jansen@rug.nl>

### References

Y. Li, R. Breitling and R.C. Jansen. Generalizing genetical
genomics: the added value from environmental perturbation, Trends Genet
(2008) 24:518-524.   
Y. Li, M. Swertz, G. Vera, J. Fu, R. Breitling, and R.C. Jansen. designGG:
An R-package and Web tool for the optimal design of genetical genomics
experiments. (submitted)   
http://gbic.biol.rug.nl/designGG

### See Also

`initialDesign`

---

[Package *designGG* version 1.0-02 Index]
