# Supplementary material for: designGG: an R-package and web tool for the optimal design of genetical genomics experiments
Source: BMC Bioinformatics. 2009 Jun 18;10:188. doi: 10.1186/1471-2105-10-188 (PMC2706229; doi:10.1186/1471-2105-10-188)
Supplement: Additional file 1 — designGG: an R-package for the optimal design of genetical genomics experiments. DesignGG aims at finding an optimal design of genetical genomics experiments which maximize the power and resolution of detecting genetic, environmental and interaction effects. This will help to achieve high power and more accurate estimates of the effects of interesting factors, and thus yield a more reliable biological interpretation of data. [file 1471-2105-10-188-S1.zip › designGG/html/conditionCombination.html]

R: Generate a matrix indicating all possible levels for environmental
factors

|  |  |
| --- | --- |
| conditionCombination {designGG} | R Documentation |

## Generate a matrix indicating all possible levels for environmental factors

### Description

Generate a matrix indicating all possible levels for environmental
factors with dimension nConditions $times$ nEnvFactors.
This is a subfunction needed for `designScore`, but is not directly used.

### Usage

```
  conditionCombination( nEnvFactors, nLevels, Level, envFactorNames )
```

### Arguments

|  |  |
| --- | --- |
| `nEnvFactors` | number of environmental factors, an integer bewteen 1 and 3. When `nEnvFactors` is 1 and the number of levels for the enviromental factor (`nLevels`) is 1, there is one condition in the experiment (i.e. no enviromental perturbation) and thus only genetic factor will be considered in the algorithm. When `nEnvFactors` is 1 and nLevels is larger than 1 or `nEnvFactors` is larger than 1, all main factor(s) and interacting facotr(s) will be included. Examples: If there is a temperature perturbation, then `nEnvFactors` is 1; If there is both temperature and drug treatment perturbation, then `nEnvFactors` is 2. |
| `nLevels` | number of levels for each factor, a vector with each component being integer. The length should be equal to `nEnvFactors`. |
| `Level` | a list which specifies the levels for each factor in the experiment. There are in total `nEnvFactors` elements in the list and each element correspsonds to certain envrironmental factor. The element is a vector describing all levels of the environmental factor. Default setting for the level of each factor is 1, 2, ...., nLevels[i]. (Here nLevels[i] is the *i*th element of nLevels, which tells the total number of levels for *i* environmental factor). |
| `envFactorNames` | a vector with names for all environmental factor(s). For example, for an experiment with two environmental factors of temperature and drug treatment: `envFactorNames <- c( "Temperature", "Dosage" )`   Default = `NULL`, then the output will use `"F1"` and `"F2"` to indicate the environmental factors. |

### Details

Currently this function works only when `nEnvFactors` is between 1 and 3.

### Value

A matrix with dimension of nConditions $times$ nEnvFactors.
Each element in the matrix indicates the levels of corresponding environmental
factor.

### Author(s)

Yang Li <yang.li@rug.nl>, Gonzalo Vera <gonzalo.vera.rodriguez@gmail.com>   
Rainer Breitling <r.breitling@rug.nl>, Ritsert Jansen <r.c.jansen@rug.nl>

### References

Y. Li, R. Breitling and R.C. Jansen. Generalizing genetical
genomics: the added value from environmental perturbation, Trends Genet
(2008) 24:518-524.   
Y. Li, M. Swertz, G. Vera, J. Fu, R. Breitling, and R.C. Jansen. designGG:
An R-package and Web tool for the optimal design of genetical genomics
experiments. (submitted)   
http://gbic.biol.rug.nl/designGG

### See Also

`designScore`

---

[Package *designGG* version 1.0-02 Index]
