# Supplementary material for: designGG: an R-package and web tool for the optimal design of genetical genomics experiments
Source: BMC Bioinformatics. 2009 Jun 18;10:188. doi: 10.1186/1471-2105-10-188 (PMC2706229; doi:10.1186/1471-2105-10-188)
Supplement: Additional file 1 — designGG: an R-package for the optimal design of genetical genomics experiments. DesignGG aims at finding an optimal design of genetical genomics experiments which maximize the power and resolution of detecting genetic, environmental and interaction effects. This will help to achieve high power and more accurate estimates of the effects of interesting factors, and thus yield a more reliable biological interpretation of data. [file 1471-2105-10-188-S1.zip › designGG/html/conditionLevel.html]

R: Levels of all environmental factors

|  |  |
| --- | --- |
| conditionLevel {designGG} | R Documentation |

## Levels of all environmental factors

### Description

Describe the levels of all environmental factors for each RIL/strain in the
experiment.  
This is a subfunction needed for `designScore`, but is not directly used.

### Usage

```
    conditionLevel( array.allocation, condition.allocation, 
                    condition.combination,nEnvFactors )
```

### Arguments

|  |  |
| --- | --- |
| `array.allocation` | a matrix with nArray rows and nRIL columns. Elements of 1/0 indicates this RIL (or strain) is/not selected for this array. |
| `condition.allocation` | a matrix with nCondition rows and nRIL columns. Elements of 1/0 indicates this RIL (or strain) is/not selected for this condition. |
| `condition.combination` | a matrix indicating all possible levels for environmental factors, with dimension of nConditions by nEnvFactors. |
| `nEnvFactors` | number of environmental factors, an integer bewteen 1 and 3. When `nEnvFactors` is 1 and nLevels is 1, there is one condition in the experiment (i.e. no enviromental perturbation) and thus only genetic factor will be considered in the algorithm. When `nEnvFactors` is 1 and nLevels is larger than 1 or `nEnvFactors` is larger than 1, all main factor(s) and interacting facotr(s) will be included. |

### Details

For single-channel experiment, `array.allocation` is `NULL`. Then the `conditionLevel` is
decided by `condition.allocation`.
For dual-channel experiment, `array.allocation` decides which RILs are selected and
then the `condition.allocation` indicates which condition this RIL will be put in for the
experiment.

### Value

A matrix with dimension of nRILs by nEnvFactors, each element indicates the
level of a certain environmental factor to which the RIL (or strain) is exposed in the
experiment.

### Author(s)

Yang Li <yang.li@rug.nl>, Gonzalo Vera <gonzalo.vera.rodriguez@gmail.com>   
Rainer Breitling <r.breitling@rug.nl>, Ritsert Jansen <r.c.jansen@rug.nl>

### References

Y. Li, R. Breitling and R.C. Jansen. Generalizing genetical
genomics: the added value from environmental perturbation, Trends Genet
(2008) 24:518-524.   
Y. Li, M. Swertz, G. Vera, J. Fu, R. Breitling, and R.C. Jansen. designGG:
An R-package and Web tool for the optimal design of genetical genomics
experiments. (submitted)   
http://gbic.biol.rug.nl/designGG

### See Also

`designScore`, `conditionCombination`

---

[Package *designGG* version 1.0-02 Index]
