# Supplementary material for: designGG: an R-package and web tool for the optimal design of genetical genomics experiments
Source: BMC Bioinformatics. 2009 Jun 18;10:188. doi: 10.1186/1471-2105-10-188 (PMC2706229; doi:10.1186/1471-2105-10-188)
Supplement: Additional file 1 — designGG: an R-package for the optimal design of genetical genomics experiments. DesignGG aims at finding an optimal design of genetical genomics experiments which maximize the power and resolution of detecting genetic, environmental and interaction effects. This will help to achieve high power and more accurate estimates of the effects of interesting factors, and thus yield a more reliable biological interpretation of data. [file 1471-2105-10-188-S1.zip › designGG/html/conditionUpdate.html]

R: Update condition allocation

|  |  |
| --- | --- |
| conditionUpdate {designGG} | R Documentation |

## Update condition allocation

### Description

Update the allocation of samples onto different conditions. This is a subfunction needed for `updateDesign`, but is not directly used.

### Usage

```
    conditionUpdate( condition.allocation, nTuple )
```

### Arguments

|  |  |
| --- | --- |
| `condition.allocation` | a matrix with nCondition rows and nRIL columns. elements of 1/0 indicate this RIL (or strain) is/not selected for this condition. |
| `nTuple` | average number of RILs (or strains) to be assigned onto each condition   `nTuple` should be a real number which is larger than 1.  if `nTuple` < 1, the algorithm will stop and show the message,   `warning: "The number slides is too less to perform the experiment."` |

### Details

This function will be used both in single and dual channel experiment
design.

### Value

An updated `condition.allocation` table.

### Author(s)

Yang Li <yang.li@rug.nl>, Gonzalo Vera <gonzalo.vera.rodriguez@gmail.com>   
Rainer Breitling <r.breitling@rug.nl>, Ritsert Jansen <r.c.jansen@rug.nl>

### References

Y. Li, R. Breitling and R.C. Jansen. Generalizing genetical
genomics: the added value from environmental perturbation, Trends Genet
(2008) 24:518-524.   
Y. Li, M. Swertz, G. Vera, J. Fu, R. Breitling, and R.C. Jansen. designGG:
An R-package and Web tool for the optimal design of genetical genomics
experiments. (submitted)   
http://gbic.biol.rug.nl/designGG

### See Also

`arrayUpdate`, `designGG`

---

[Package *designGG* version 1.0-02 Index]
