# Supplementary material for: designGG: an R-package and web tool for the optimal design of genetical genomics experiments
Source: BMC Bioinformatics. 2009 Jun 18;10:188. doi: 10.1186/1471-2105-10-188 (PMC2706229; doi:10.1186/1471-2105-10-188)
Supplement: Additional file 1 — designGG: an R-package for the optimal design of genetical genomics experiments. DesignGG aims at finding an optimal design of genetical genomics experiments which maximize the power and resolution of detecting genetic, environmental and interaction effects. This will help to achieve high power and more accurate estimates of the effects of interesting factors, and thus yield a more reliable biological interpretation of data. [file 1471-2105-10-188-S1.zip › designGG/html/designGG.html]

R: Optimal design for genetical genomics experiments

|  |  |
| --- | --- |
| designGG {designGG} | R Documentation |

## Optimal design for genetical genomics experiments

### Description

Main function to search and display A- and D- optimal designs for single- or
two-channel genetical genomics experiments. Simulated annealing or Metropolis
Hastings used to find the best design.

### Usage

```
  designGG <- function( genotype, nSlides, nTuple, nEnvFactors, nLevels,
                     Level=NULL, bTwoColorArray=T, initial=NULL, weight=1,
                     region=NULL, optimality="A", method="SA", nIterations=3000,
                     n.search=2, endTemp=1e-10, startTemp=1, maxTempStep=0.9,
                     plotScores=T, directory=NULL, fileName=NULL, 
                     envFactorNames=NULL, writingProcess=T )
```

### Arguments

|  |  |
| --- | --- |
| `genotype` | genotype data: a nMarker-by-nRILs matrix with two allels being 0 and 1 (or A and B) or three allels being 0, 0.5 and 1 (or, A, H, and B), where 0.5 (or H) represents heterozygous allele. |
| `nSlides` | total number of slides available for the experiment. |
| `nTuple` | average number of RILs (or strains) to be assigned onto each condition.   `nTuple` should be a real number which is larger than 1.  If `nTuple` < 1, the algorithm will stop and show the message,   `warning: "The number of slides is too small to perform the experiment."` |
| `nEnvFactors` | number of environmental factors, an integer bewteen 1 and 3. When `nEnvFactors` is 1 and the number of levels for the enviromental factor (`nLevels`)is 1, there is one condition in the experiment (i.e. no enviromental perturbation) and thus only genetic factor will be considered in the algorithm. When `nEnvFactors` is 1 and nLevels is larger than 1 or `nEnvFactors` is larger than 1, all main factor(s) and interacting facotr(s) will be included. Examples: If there is a temperature perturbation, then `nEnvFactors` is 1; If there is both temperature and drug treatment perturbation, then `nEnvFactors` is 2. |
| `nLevels` | number of levels for each factor, a vector with each component being an integer. The length of it should equal `nEnvFactors`. |
| `Level` | a list which specifies the levels for each factor in the experiment. There are in total `nEnvFactors` elements in the list and each element corresponds to a certain environmental factor. The element is a vector describing all levels of the environmental factor. default setting for the level of each factor is 1, 2, ...., nlevels[i]. (Here nLevels[i] is the *i*th element of nLevels, which gives the total number of levels for *i* environmental factor). |
| `bTwoColorArray` | binary variable indicating experiment type:   `bTwoColorArray <- T` #for dual channel experiment   `bTwoColorArray <- F` #for single channel experiment |
| `initial` | the starting design matrix for the algorithm. If specified, this should be a list with 2 matrices:   `condition.allocation`: allocate RILs (or strains) into different conditional (nrow = nCondition, ncol= nRILs)   `array.allocation`: pair RILs (or strains) into sldies (nrow = nSlide, ncol = nRILs)   However, the algorithm does not require that a starting matrix is specified. Default = `NULL`. |
| `weight` | a vector with length of `variableNumber` which is calculated from function `variableNumber`. Default = 1 (which means the parameters to be estimated are all equally important during optimization). See details below. |
| `region` | genome region of biological interest. Default = `NULL` (which means the entire genome will considered). |
| `optimality` | type of optimality, i.e. "A" (A-optimality) or "D" (D-optimality). A-optimality minimizes $Trace((X'X)^{-1})$, which corresponds to minimum average variance of the parameter estimates. D-optimality minimizes $det(X'X)^{-1}$, which corresponds to minimum generalized variance of the parameter estimates. |
| `method` | method for searching for an optimal design. "SA" uses simulated annealing. "MH" uses Metropolis Hasting. Default = "SA". |
| `nIterations` | number of iterations of the simulated annealing method. Default = 3000. |
| `n.search` | number of times for simulated annealing optimaization with different initial design, default = 2. Here it is suggested to be between 1 and 5. It should not to be too large because of the reaching computational burden. |
| `endTemp` | ending temperature of simulated annealing process. An important optimization parameter. Default = $1e^{-10}$. |
| `startTemp` | starting temperature of simulated annealing process. Default = 1. |
| `maxTempStep` | maximum temperature decreasing step for simulated annealing process. The parameter ensures that the multiplicative cooling factor is not smaller than that. If `nIterations` is too small, the preferred final temperature (endTemp) may not be reached. See Wit and McClure (2004) for details. Default = 0.9. |
| `plotScores` | If `TRUE` (default) it produces a plot of the optimazation by SA using the function plotAllScores. |
| `directory` | It tells where the resulting optimal design tables are to be stored. If `NULL` (default), it will take currect working directory. |
| `fileName` | the final optimal design table(s) in `csv` format and a plot (in `png` format) of all scores during SA process (if `plotScores` = T) will be produced. The users can specify the table and plot name by setting `fileName`. If `NULL` (default) it produces files starting with `"myDesignGG"`. |
| `envFactorNames` | a vector with names for all environmental factor(s). For example, for the experiment with two environmental factors of temperature and drug treatment: `envFactorNames <- c( "Temperature", "Dosage" )`    Default = `NULL`, then the output will use `"F1"` and `"F2"` to indicate the environmental factors. |
| `writingProcess` | If TRUE, it prints how much computation work has been finished in a file called `"processing.txt"`. Default = `TRUE`. |

### Details

Given the genetic information of samples available for the experiment
(genotype) and the information about experimental settings (`nEnvFactors`,
`nSlides`,`nLevels` etc.), the algorithm searches for an A-optimal or D-optimal
(see `optimality`) using simulated annealing (see `method`). A plot of
the scores at each iterations can also be given using the `plotAllScores`
function.   
It also contains a number of the arguments:  
`region` is used to specify the
genome region that are of major interest to experimenters.   
`weight` is used to define
the weight of genetic and environmental factors, and interaction terms. Prior
knowledge about expected effect sizes of interesting factors can also be
incorporated as `weight` parameters for the algorithm. The weight is
inversely proportional to the expected effect size of the corresponding parameter.
Example parameter settings:
Suppose to design an experiment with two environmental factors (F1, F2) and
there are two diffferent levels for each environment. The levels are 16
and 24 for F1, and 5 and 10 for F2. Thus the following command can be used:  
 `nEnvFactors <- 2`    
 `nLevels <- c ( 2, 2 )`   
 `levels <- list ( c(16, 24), c(5, 10) )`    
The length of parameter `weight` is dependent on the number of environmental
factors:   
When `nEnvFactor` = 0,   
`weight` is 1 as there is only one parameter of interest (genotype).  
When `nEnvFactor` = 1,   
`weight` = c( $w\_{Q}$, $w\_{F1}$, $w\_{QF1}$ )   
When `nEnvFactor` = 2,   
`weight` = c( $w\_{Q}$, $w\_{F1}$, $w\_{F2}$, $w\_{QF1}$, $w\_{QF2}$, $w\_{F1F2}$, $w\_{QF1F2}$)   
When `nEnvFactor` = 3,   
`weight` = c( $w\_{Q}$, $w\_{F1}$, $w\_{F2}$, $w\_{F2}$,
$w\_{QF1}$, $w\_{QF2}$, $w\_{QF3}$, $w\_{F1F2}$, $w\_{F1F3}$, $w\_{F2F3}$,
$w\_{QF1F2}$, $w\_{QF1F3}$, $w\_{QF2F3}$, $w\_{QF1F2F3}$ )   
Here $w\_{Q}$ represents the weight for genotype effect, $w\_{F1}$ represent the
weight for F1 effect and $w\_{QF1}$ represent the weight for interaction between
genotype and F1 effect, etc.   
It should be noted that the simulated annealing algorithm might find a
locally and not globally optimal design. Running the optimization process
multiple times is recommended. When `nSearch` > 1, the simulated annealing
optimization will be run nSearch times, each run starts with a different
initial design and will provide a (near-)optimal design. If the optimization
problem is simple, all runs will converge to the same (optimal) design.
Otherwise, the best one among all near-optimal designs will be selected as
the output of the function. One can run the algorithm multiple times with
`nSearch` = 1 to review a few (near-)optimal designs.

### Value

An array design table (arrayDesign.csv) and a condition design table (
conditionDesign.csv) are generated.

### Author(s)

Yang Li <yang.li@rug.nl>, Gonzalo Vera <gonzalo.vera.rodriguez@gmail.com>   
Rainer Breitling <r.breitling@rug.nl>, Ritsert Jansen <r.c.jansen@rug.nl>

### References

Y. Li, M. Swertz, G. Vera, J. Fu, R. Breitling, and R.C. Jansen. designGG:
An R-package and Web tool for the optimal design of genetical genomics
experiments. (submitted)   
http://gbic.biol.rug.nl/designGG   
Y. Li, R. Breitling and R.C. Jansen. Generalizing genetical
genomics: the added value from environmental perturbation, Trends Genet
(2008) 24:518-524.   
E. Wit and J. McClure. Statistics for Microarrays: Design, Analysis
and Inference. (2004) Chichester: Wiley.

### See Also

`initialDesign`, `designScore`,
`updateDesign`, `acceptanceProbability`,   
`experimentDesignTable`, `plotAllScores`,  
`exampleArrayDesignTable`,`exampleConditionDesignTable`,

### Examples

```
  library(designGG)
  #load genotype data
  data(genotype)
  #Example:  single-channel experiment with 2 environmental factors,
  #each with 2 level, and there will be four samples per condition(nTuple=4).
  optimalDesign <- designGG ( genotype, nSlides=NULL, nTuple=4, nEnvFactors=2,
                        nLevels=c(2,2),Level=list(c(16,24),c(5,10)),  bTwoColorArray=F,
                        initial=NULL, weight=1, region=seq(1,20), optimality="A", 
                        method="SA", nIterations=100, n.search=2, endTemp=1e-10,
                        startTemp=1, maxTempStep=0.9, plotScores=T, 
                        directory=NULL, fileName=NULL, envFactorNames=NULL, 
                        writingProcess=F )
  #Example 2:  dual-channel experiment with 2 environmental factors,
  #each with 2 level. There are 50 slides available.
  optimalDesign <- designGG ( genotype, nSlides=50, nTuple=NULL, nEnvFactors=2,
                        nLevels=c(2,2),Level=list(c(16,24),c(5,10)),  bTwoColorArray=T,
                        initial=NULL, weight=1, region=seq(1,20), optimality="A", 
                        method="SA", nIterations=100, n.search=2, endTemp=1e-10,
                        startTemp=1, maxTempStep=0.9, plotScores=T, 
                        directory=NULL, fileName=NULL, envFactorNames=NULL, 
                        writingProcess=F )
  #result
  optimalDesign$arrayDesign
  optimalDesign$conditionDesign
  plotAllScores(optimalDesign$plot.obj)
  
  #Use the following commands to see example output tables: 
  data(exampleArrayDesignTable)
  exampleArrayDesignTable
  data(exampleConditionDesignTable)
  exampleConditionDesignTable
```

---

[Package *designGG* version 1.0-02 Index]
