# Supplementary material for: designGG: an R-package and web tool for the optimal design of genetical genomics experiments
Source: BMC Bioinformatics. 2009 Jun 18;10:188. doi: 10.1186/1471-2105-10-188 (PMC2706229; doi:10.1186/1471-2105-10-188)
Supplement: Additional file 1 — designGG: an R-package for the optimal design of genetical genomics experiments. DesignGG aims at finding an optimal design of genetical genomics experiments which maximize the power and resolution of detecting genetic, environmental and interaction effects. This will help to achieve high power and more accurate estimates of the effects of interesting factors, and thus yield a more reliable biological interpretation of data. [file 1471-2105-10-188-S1.zip › designGG/html/designScore.html]

R: Calculate the A- or D- optimality score based on current experimental design

|  |  |
| --- | --- |
| designScore {designGG} | R Documentation |

## Calculate the A- or D- optimality score based on current experimental design

### Description

According to the current experimental design, the Fisher information matrix
is obtained and then either the A- or D- optimality score is computed.

### Usage

```
  designScore( genotype, array.allocation, condition.allocation, 
               nEnvFactors, nLevels, Level, nConditions, weight=1, 
               optimality="A", bTwoColorArray, envFactorNames)
```

### Arguments

|  |  |
| --- | --- |
| `genotype` | genotype data: a nMarker-by-nRILs matrix with two allels being 0 and 1 (or A and B) or three allels being 0, 0.5 and 1 (or, A, H, and B), where 0.5 (or H) represents heterozygous allele. |
| `array.allocation` | matrix with nArray rows and nRIL columns. Elements of 1/0 indicate this RIL (or strains) is/not selected for this array. |
| `condition.allocation` | matrix with nCondition rows and nRIL columns. Elements of 1/0 indicate this RIL (or strains) is/not selected for this condition. |
| `nEnvFactors` | number of environmental factors, an integer bewteen 1 and 3. When `nEnvFactors` is 1 and the number of levels for the enviromental factor (`nLevels`)is 1, there is one condition in the experiment (i.e. no enviromental perturbation) and thus only genetic factor will be considered in the algorithm. When `nEnvFactors` is 1 and nLevels is larger than 1 or `nEnvFactors` is larger than 1, all main factor(s) and interacting facotr(s) will be included. Examples: If there is a temperature perturbation, then `nEnvFactors` is 1; If there is both temperature and drug treatment perturbation, then `nEnvFactors` is 2. |
| `nLevels` | number of levels for each factor, a vector with each component being an integer. The length of it should equal `nEnvFactors`. |
| `Level` | a list which specifies the levels for each factor in the experiment. There are in total `nEnvFactors` elements in the list and each element correpsond to certain envrironmental factor. The emlemet is a vector describing all levels of the environmental factor. default setting for the level of each factor is 1, 2, ... nLevels[i]. (Here nLevels[i] is the *i*th element of nLevels, which gives the total number of levels for *i* environmental factor). |
| `nConditions` | number of all possible combination of all environmental factors. |
| `weight` | a vector with length of variableNumber which is calculated from function `variableNumber`. Default = 1 (which means the parameters to be estimated are equally important during optimization.) |
| `optimality` | type of optimality, i.e. "A" (A-optimality) or "D" (D-optimality). A-optimality minimizes $Trace((X'X)^{-1})$, which corresponds to minimum average variance of the parameter estimates. D-optimality minimizes $det(X'X)^{-1}$, which corresponds to minimum generalized variance of the parameter estimates. |
| `bTwoColorArray` | binary variable indicating experiment type:   `bTwoColorArray <- T` #for dual channel experiment   `bTwoColorArray <- F` #for single channel experiment |
| `envFactorNames` | a vector with names for all environmental factor(s). For example, for the experiment with two environmental factors of temperature and drug treatment: `envFactorNames <- c( "Temperature", "Dosage" )`   Default = `NULL`, then the output will use "F1" and "F2" to indicate the environmental factors. |

### Details

Example parameter settings:  
Suppose to design an experiment with two environmental factors (F1, F2) and
there are two diffferent levels for each environment. The levels are 16
and 24 for F1, and 5 anf 10 for F2. Thus the following command can be used:  
`nEnvFactors <- 2`  
`nLevels <- c ( 2, 2 )`  
`levels <- list ( c(16, 24), c(5, 10) )`  
The length of parameter `weight` is dependent on the number of environmental
factors:   
When `nEnvFactor` = 0,   
`weight` is 1 as there is only one parameter of interest (genotype).  
When `nEnvFactor` = 1,   
`weight` = c( $w\_{Q}$, $w\_{F1}$, $w\_{QF1}$ )   
When `nEnvFactor` = 2,   
`weight` = c( $w\_{Q}$, $w\_{F1}$, $w\_{F2}$, $w\_{QF1}$, $w\_{QF2}$, $w\_{F1F2}$, $w\_{QF1F2}$)   
When `nEnvFactor` = 3,   
`weight` = c( $w\_{Q}$, $w\_{F1}$, $w\_{F2}$, $w\_{F2}$,
$w\_{QF1}$, $w\_{QF2}$, $w\_{QF3}$, $w\_{F1F2}$, $w\_{F1F3}$, $w\_{F2F3}$,
$w\_{QF1F2}$, $w\_{QF1F3}$, $w\_{QF2F3}$, $w\_{QF1F2F3}$ )   
Here $w\_{Q}$ represents the weight for genotype effect, $w\_{F1}$ represent the
weight for F1 effect and $w\_{QF1}$ represent the weight for interaction between
genotype and F1 effect, etc.

### Value

The score is defined as the "double" sum of the variances, summed over all
parameters and over all markers.

### Author(s)

Yang Li <yang.li@rug.nl>, Gonzalo Vera <gonzalo.vera.rodriguez@gmail.com>   
Rainer Breitling <r.breitling@rug.nl>, Ritsert Jansen <r.c.jansen@rug.nl>

### References

Y. Li, M. Swertz, G. Vera, J. Fu, R. Breitling, and R.C. Jansen. designGG:
An R-package and Web tool for the optimal design of genetical genomics
experiments. (submitted)   
http://gbic.biol.rug.nl/designGG   
Y. Li, R. Breitling and R.C. Jansen. Generalizing genetical
genomics: the added value from environmental perturbation, Trends Genet
(2008) 24:518-524.   
E. Wit and J. McClure. Statistics for Microarrays: Design, Analysis
and Inference. (2004) Chichester: Wiley.

### See Also

`designGG`

---

[Package *designGG* version 1.0-02 Index]
