# Supplementary material for: designGG: an R-package and web tool for the optimal design of genetical genomics experiments
Source: BMC Bioinformatics. 2009 Jun 18;10:188. doi: 10.1186/1471-2105-10-188 (PMC2706229; doi:10.1186/1471-2105-10-188)
Supplement: Additional file 1 — designGG: an R-package for the optimal design of genetical genomics experiments. DesignGG aims at finding an optimal design of genetical genomics experiments which maximize the power and resolution of detecting genetic, environmental and interaction effects. This will help to achieve high power and more accurate estimates of the effects of interesting factors, and thus yield a more reliable biological interpretation of data. [file 1471-2105-10-188-S1.zip › designGG/html/exampleArrayDesignTable.html]

R: Example output of ArrayDesignTable data

|  |  |
| --- | --- |
| exampleArrayDesignTable {designGG} | R Documentation |

## Example output of ArrayDesignTable data

### Description

`exampleArrayDesignTable`: Example data of `exampleArrayDesignTable` for a
hypothetical dual-channel microarray experiment in which there are 100 strains
(e.g. recombinant inbred lines) and 27 arrays available. Two environmental factors
(temperature and cell type) are considered in this experiment. There are three
levels for temperature (15, 24 and 29) and four levels for cell types (A,B,C,D).
This table tells how to pair samples into arrays.

```
data(exampleArrayDesignTable) 
exampleArrayDesignTable[1:5,]
```

|  |  |  |
| --- | --- | --- |
|  | Channel 1 | Channel 2 |
| array1 | Strain28 | Strain92 |
| array2 | Strain70 | Strain47 |
| array3 | Strain22 | Strain89 |
| array4 | Strain45 | Strain15 |
| array5 | Strain52 | Strain41 |

### Usage

```
data(exampleArrayDesignTable)
```

### Format

`exampleArrayDesignTable`: 27 arrays by two channels.

### Author(s)

Yang Li <yang.li@rug.nl>, Gonzalo Vera <gonzalo.vera.rodriguez@gmail.com>   
Rainer Breitling <r.breitling@rug.nl>, Ritsert Jansen <r.c.jansen@rug.nl>

### Examples

```
  ##load the data
  data(exampleArrayDesignTable)
  
  ##view part of the the data
  exampleArrayDesignTable[1:5,]
```

---

[Package *designGG* version 1.0-02 Index]
