# Supplementary material for: designGG: an R-package and web tool for the optimal design of genetical genomics experiments
Source: BMC Bioinformatics. 2009 Jun 18;10:188. doi: 10.1186/1471-2105-10-188 (PMC2706229; doi:10.1186/1471-2105-10-188)
Supplement: Additional file 1 — designGG: an R-package for the optimal design of genetical genomics experiments. DesignGG aims at finding an optimal design of genetical genomics experiments which maximize the power and resolution of detecting genetic, environmental and interaction effects. This will help to achieve high power and more accurate estimates of the effects of interesting factors, and thus yield a more reliable biological interpretation of data. [file 1471-2105-10-188-S1.zip › designGG/html/examplePlotObj.html]

R: Example PlotObj data

|  |  |
| --- | --- |
| examplePlotObj {designGG} | R Documentation |

## Example PlotObj data

### Description

`examplePlotObj`:
Example data of `examplePlotObj` for plot all scores and cooling at each
iteration during simulated annealing process.

```
data(examplePlotObj)
plotAllScores(examplePlotObj)
```

### Usage

```
data(examplePlotObj)
```

### Format

`examplePlotObj`: a list which contains the following elements:
(1) scores (2) cooling (3) startTemp (4) temperature
(5) temperature.step (6) nIterations (7) optimality.

### Author(s)

Yang Li <yang.li@rug.nl>, Gonzalo Vera <gonzalo.vera.rodriguez@gmail.com>   
Rainer Breitling <r.breitling@rug.nl>, Ritsert Jansen <r.c.jansen@rug.nl>

---

[Package *designGG* version 1.0-02 Index]
