# Supplementary material for: designGG: an R-package and web tool for the optimal design of genetical genomics experiments
Source: BMC Bioinformatics. 2009 Jun 18;10:188. doi: 10.1186/1471-2105-10-188 (PMC2706229; doi:10.1186/1471-2105-10-188)
Supplement: Additional file 1 — designGG: an R-package for the optimal design of genetical genomics experiments. DesignGG aims at finding an optimal design of genetical genomics experiments which maximize the power and resolution of detecting genetic, environmental and interaction effects. This will help to achieve high power and more accurate estimates of the effects of interesting factors, and thus yield a more reliable biological interpretation of data. [file 1471-2105-10-188-S1.zip › designGG/html/experimentDesignTable.html]

R: Make experiment table based design matrix

|  |  |
| --- | --- |
| experimentDesignTable {designGG} | R Documentation |

## Make experiment table based design matrix

### Description

This function generates two `.csv` files which descibe how samples are allocated
samples into different conditions and paired on arrays.

### Usage

```
  experimentDesignTable( array.allocation, condition.allocation, 
                         nEnvFactors, nLevels, Level, fileName,envFactorNames,
                         directory )
```

### Arguments

|  |  |
| --- | --- |
| `array.allocation` | matrix with nArray rows and nRIL columns. Elements of 1/0 indicate this RIL is/not selected for this array. |
| `condition.allocation` | matrix with nCondition rows and nRIL columns. Elements of 1/0 indicate this RIL is/not selected for this condition. |
| `nEnvFactors` | number of environmental factors, an integer bewteen 1 and 3. When `nEnvFactors` is 1 and the number of levels for the enviromental factor (`nLevels`)is 1, there is one condition in the experiment (i.e. no enviromental perturbation) and thus only genetic factor will be considered in the algorithm. When `nEnvFactors` is 1 and nLevels is larger than 1 or `nEnvFactors` is larger than 1, all main factor(s) and interacting facotr(s) will be included. Examples: If there is a temperature perturbation, then `nEnvFactors` is 1; If there is both temperature and drug treatment perturbation, then `nEnvFactors` is 2. |
| `nLevels` | number of levels for each factor, a vector with each component being integer. The length of it should equal `nEnvFactors`. |
| `Level` | a list which specifies the levels for each factor in the experiment. There are in total `nEnvFactors` elements in the list and each element correpsond to certain envrironmental factor. The emlemet is a vector describing all levels of the environmental factor. default setting for the level of each factor is 1, 2, ...nLevels[i]. (Here nLevels[i] is the *i*th element of nLevels, which gives the total number of levels for *i* environmental facotor). |
| `fileName` | the final optimal design table(s) in `csv` format and a plot (in `png` format) of the all scores during SA process (if `plotScores` = T) will be produced. The users can specify the table and plot name by setting `fileName`. If `NULL` (default) it produces files starting with `"myDesignGG"`. |
| `envFactorNames` | a vector with names for all environmental factor(s). For example, for the experiment with two environmental factors of temperature and drug treatment: `envFactorNames <- c( "Temperature", "Dosage" )`    Default = `NULL`, then the output will use "F1" and "F2" to indicate the environmental factors. |
| `directory` | It tells where the resulting optimal design tables are to be stored. If `NULL` (default), it will use the currect working directory. |

### Details

Based on `nEnvFactors` and `nLevels`, `nConditions` is calculated.

### Value

Two tables report the results: table "pair design" which is only used for
two-channel experiments and describes how samples are paired together on the
slide (e.g. microarray chip), and table "envi-ronment design" which is used
when there are more environments evolved in the experiment. With these two
tables, the experimenters can set up the environmental treatment and
follow-up profiling measurement.   
Examples:   
1. conditionDesign.csv   

|  |  |  |  |  |  |  |  |
| --- | --- | --- | --- | --- | --- | --- | --- |
|  | Temperature | Cell Type |  |  | Selected Samples |  |  |
| condition1 | 15 | A | RIL28 | RIL81 | RIL18 | RIL61 |  |
| condition2 | 24 | A | RIL72 | RIL40 | RIL83 | RIL44 | RIL10 |
| condition3 | 29 | A | RIL22 | RIL89 | RIL3 | RIL30 | RIL58 |
| condition4 | 15 | B | RIL70 | RIL47 | RIL4 | RIL59 |  |
| condition5 | 24 | B | RIL93 | RIL97 | RIL49 | RIL14 |  |

2. arrayDesign.csv   

|  |  |  |
| --- | --- | --- |
|  | Channel 1 | Channel 2 |
| array1 | RIL28 | RIL92 |
| array2 | RIL70 | RIL47 |
| array3 | RIL22 | RIL89 |
| array4 | RIL45 | RIL15 |
| array5 | RIL52 | RIL41 |

### Note

The optimal design results are described in two tables. One is called "array
design" which is only used for two-channel experiments. It describes how
samples are paired together on the slide (e.g. microarray chip). The other
table is called "condition design" which is used when there is more than
one environmental factor involved in the experiment. Each cell in condition
design table represents a combination of different levels of environmental
factors and the selected sample names (e.g. RIL names) for this condition
are shown. Based on these two tables, the experimenters can set up the
environmental treatment and follow-up profiling measurement.

### Author(s)

Yang Li <yang.li@rug.nl>, Gonzalo Vera <gonzalo.vera.rodriguez@gmail.com>   
Rainer Breitling <r.breitling@rug.nl>, Ritsert Jansen <r.c.jansen@rug.nl>

### References

Y. Li, R. Breitling and R.C. Jansen. Generalizing genetical
genomics: the added value from environmental perturbation, Trends Genet
(2008) 24:518-524.   
Y. Li, M. Swertz, G. Vera, J. Fu, R. Breitling, and R.C. Jansen. designGG:
An R-package and Web tool for the optimal design of genetical genomics
experiments. (submitted)   
http://gbic.biol.rug.nl/designGG

### See Also

`designGG`, `exampleArrayDesignTable`, `exampleConditionDesignTable`

---

[Package *designGG* version 1.0-02 Index]
