# Supplementary material for: designGG: an R-package and web tool for the optimal design of genetical genomics experiments
Source: BMC Bioinformatics. 2009 Jun 18;10:188. doi: 10.1186/1471-2105-10-188 (PMC2706229; doi:10.1186/1471-2105-10-188)
Supplement: Additional file 1 — designGG: an R-package for the optimal design of genetical genomics experiments. DesignGG aims at finding an optimal design of genetical genomics experiments which maximize the power and resolution of detecting genetic, environmental and interaction effects. This will help to achieve high power and more accurate estimates of the effects of interesting factors, and thus yield a more reliable biological interpretation of data. [file 1471-2105-10-188-S1.zip › designGG/html/genotype.html]

R: Example genotype data

|  |  |
| --- | --- |
| genotype {designGG} | R Documentation |

## Example genotype data

### Description

`genotype`: example data of `genotypes` for each marker (rownames)
and 100 strains such as recombinant inbred lines (RIL) (columnnames),
with numeric values 1 and 0 (or A and B).

```
data(genotypes)
genotypes[1:5,1:5]
```

  

|  |  |  |  |  |  |
| --- | --- | --- | --- | --- | --- |
|  | Strain1 | Strain2 | Strain3 | Strain4 | Strain5 |
| C1M1 | 1 | 0 | 0 | 0 | 1 |
| C1M2 | 1 | 0 | 0 | 0 | 1 |
| C1M3 | 1 | 0 | 0 | 0 | 1 |
| C1M4 | 1 | 0 | 0 | 1 | 1 |
| C1M5 | 1 | 0 | 0 | 1 | 1 |

### Usage

```
data(genotypes)
```

### Format

`genotypes`: 120 markers by 100 samples (Strains).

### Author(s)

Yang Li <yang.li@rug.nl>, Gonzalo Vera <gonzalo.vera.rodriguez@gmail.com>   
Rainer Breitling <r.breitling@rug.nl>, Ritsert Jansen <r.c.jansen@rug.nl>

---

[Package *designGG* version 1.0-02 Index]
