# Supplementary material for: designGG: an R-package and web tool for the optimal design of genetical genomics experiments
Source: BMC Bioinformatics. 2009 Jun 18;10:188. doi: 10.1186/1471-2105-10-188 (PMC2706229; doi:10.1186/1471-2105-10-188)
Supplement: Additional file 1 — designGG: an R-package for the optimal design of genetical genomics experiments. DesignGG aims at finding an optimal design of genetical genomics experiments which maximize the power and resolution of detecting genetic, environmental and interaction effects. This will help to achieve high power and more accurate estimates of the effects of interesting factors, and thus yield a more reliable biological interpretation of data. [file 1471-2105-10-188-S1.zip › designGG/html/initialDesign.html]

R: Initialize an experiment design matrix

|  |  |
| --- | --- |
| initialDesign {designGG} | R Documentation |

## Initialize an experiment design matrix

### Description

Allocate RILs (or strains) into different conditional and pair RILs (or strains)
into slides.

### Usage

```
  initialDesign( genotype, nRILs, nSlides, nConditions, nTuple, 
                 bTwoColorArray )
```

### Arguments

|  |  |
| --- | --- |
| `genotype` | genotype data: a nMarker-by-nRILs matrix with two allels being 0 and 1 (or A and B) or three allels being 0, 0.5 and 1 (or, A, H, and B), where 0.5 (or H) represents heterozygous allele. |
| `nRILs` | total number of RILs ((or strains) available for the experiment. |
| `nSlides` | total number of slides available for the experiment. |
| `nConditions` | number of all possible combination of all environmental factors. |
| `nTuple` | average number of RILs (or strains) to be assigned onto each condition   `nTuple` should be a real number which is larger than 1.  if `nTuple` < 1, the algorithm will stop and shw the message below,   `warning: "The number of slides is too small to perform the experiment."` |
| `bTwoColorArray` | binary variable indicating experiment type:   `bTwoColorArray <- T` #for dual channel experiment   `bTwoColorArray <- F` #for single channel experiment |

### Details

For two-color array experiments, randomly choose a RIL (or strain) and pair it with the
genetically most different RIL (or strain) on one array.   
For one-color array experiments, array.allocation is `NULL` as there is no
need to pair samples.

### Value

a list with 2 matrices:   
`condition.allocation`: allocate RILs (or strains) into different conditional
(nCondition $times$ nRILs)   
`array.allocation`: pair RILs (or strains) into sldies
(nSlides $times$ nRILs)

### Note

This function calls `conditionAllocation` function to allocate selected RILs
(or strains) into different conditions.

### Author(s)

Yang Li <yang.li@rug.nl>, Gonzalo Vera <gonzalo.vera.rodriguez@gmail.com>   
Rainer Breitling <r.breitling@rug.nl>, Ritsert Jansen <r.c.jansen@rug.nl>

### References

Y. Li, R. Breitling and R.C. Jansen. Generalizing genetical
genomics: the added value from environmental perturbation, Trends Genet
(2008) 24:518-524.   
Y. Li, M. Swertz, G. Vera, J. Fu, R. Breitling, and R.C. Jansen. designGG:
An R-package and Web tool for the optimal design of genetical genomics
experiments. (submitted)   
http://gbic.biol.rug.nl/designGG

### See Also

`designGG`

### Examples

```
genotype <- read.table("genotype.txt")
nEnvFactors <- 2  
nLevels <- c( 2, 2 )
levels <- list ( c(16, 24), c(5, 10) )
nSlides <- 100
nTuple <- 25  
bTwoColorArray <- TRUE 
initialDesign( genotype, nRILs, nSlides, nConditions, nTuple, bTwoColorArray )
```

---

[Package *designGG* version 1.0-02 Index]
