# Supplementary material for: designGG: an R-package and web tool for the optimal design of genetical genomics experiments
Source: BMC Bioinformatics. 2009 Jun 18;10:188. doi: 10.1186/1471-2105-10-188 (PMC2706229; doi:10.1186/1471-2105-10-188)
Supplement: Additional file 1 — designGG: an R-package for the optimal design of genetical genomics experiments. DesignGG aims at finding an optimal design of genetical genomics experiments which maximize the power and resolution of detecting genetic, environmental and interaction effects. This will help to achieve high power and more accurate estimates of the effects of interesting factors, and thus yield a more reliable biological interpretation of data. [file 1471-2105-10-188-S1.zip › designGG/html/interactionLevel.html]

R: Generate levels for all interacting factors

|  |  |
| --- | --- |
| interactionLevel {designGG} | R Documentation |

## Generate levels for all interacting factors

### Description

Generate levels for all interacting factors for all RILs (or strains).
This is a subfunction needed for `designScore`, but is not directly used.

### Usage

```
  interactionLevel( genotype.level, condition.level, markerIndex, 
                    nEnvFactors )
```

### Arguments

|  |  |
| --- | --- |
| `genotype.level` | levels of genetic factor for each RIL (or strain) in the experiment. |
| `condition.level` | levels of all environmental factors for each RIL (or strain)in the experiment. |
| `markerIndex` | indicate which genome position that level of genetic factor corresponds to. |
| `nEnvFactors` | number of environmental factors, an integer bewteen 1 and 3. When `nEnvFactors` is 1 and the number of levels for the enviromental factor (`nLevels`)is 1, there is one condition in the experiment (i.e. no enviromental perturbation) and thus only genetic factor will be considered in the algorithm. When `nEnvFactors` is 1 and nLevels is larger than 1 or `nEnvFactors` is larger than 1, all main factor(s) and interacting facotr(s) will be included. Examples: If there is a temperature perturbation, then `nEnvFactors` is 1; If there is both temperature and drug treatment perturbation, then `nEnvFactors` is 2. |

### Details

`markerIndex` indicates the genome position that `genotype.level` corresponds
to.  
An experiment design is defined to be optimal over all markers if
the sum of scores, e.g. A-optimality criterion over all markers is
minimized.

### Value

a matrix with nRILs rows. The number columns depends on `nEnvFactors`.
For example:   
If `nEnvFactors` = 1, there is only one interaction term.   
If `nEnvFactors` = 2, there are three pair-wise two-way interaction terms and one
three-way interaction term.

### Author(s)

Yang Li <yang.li@rug.nl>, Gonzalo Vera <gonzalo.vera.rodriguez@gmail.com>   
Rainer Breitling <r.breitling@rug.nl>, Ritsert Jansen <r.c.jansen@rug.nl>

### References

Y. Li, R. Breitling and R.C. Jansen. Generalizing genetical
genomics: the added value from environmental perturbation, Trends Genet
(2008) 24:518-524.   
Y. Li, M. Swertz, G. Vera, J. Fu, R. Breitling, and R.C. Jansen. designGG:
An R-package and Web tool for the optimal design of genetical genomics
experiments. (submitted)   
http://gbic.biol.rug.nl/designGG

### See Also

`designScore`, `conditionLevel`

---

[Package *designGG* version 1.0-02 Index]
