# Supplementary material for: designGG: an R-package and web tool for the optimal design of genetical genomics experiments
Source: BMC Bioinformatics. 2009 Jun 18;10:188. doi: 10.1186/1471-2105-10-188 (PMC2706229; doi:10.1186/1471-2105-10-188)
Supplement: Additional file 1 — designGG: an R-package for the optimal design of genetical genomics experiments. DesignGG aims at finding an optimal design of genetical genomics experiments which maximize the power and resolution of detecting genetic, environmental and interaction effects. This will help to achieve high power and more accurate estimates of the effects of interesting factors, and thus yield a more reliable biological interpretation of data. [file 1471-2105-10-188-S1.zip › designGG/html/pairLevel.html]

R: Pair levels for paired RILs (or strains)

|  |  |
| --- | --- |
| pairLevel {designGG} | R Documentation |

## Pair levels for paired RILs (or strains)

### Description

Pair levels for two RILs (or strains) allocated into one slide (`bTwoColorArray=T`). It
is a subfunction needed for `designScore` function, but is not directly
used.

### Usage

```
  pairLevel( xxx, rilNames )
```

### Arguments

|  |  |
| --- | --- |
| `xxx` | can be `genotype.level`, `condition.level` or `interaction.level` |
| `rilNames` | names for all RILs (or strains) that have been selected for the experiment |

### Details

This function is used only for two-color array.

### Author(s)

Yang Li <yang.li@rug.nl>, Gonzalo Vera <gonzalo.vera.rodriguez@gmail.com>   
Rainer Breitling <r.breitling@rug.nl>, Ritsert Jansen <r.c.jansen@rug.nl>

### References

Y. Li, R. Breitling and R.C. Jansen. Generalizing genetical
genomics: the added value from environmental perturbation, Trends Genet
(2008) 24:518-524.   
Y. Li, M. Swertz, G. Vera, J. Fu, R. Breitling, and R.C. Jansen. designGG:
An R-package and Web tool for the optimal design of genetical genomics
experiments. (submitted)   
http://gbic.biol.rug.nl/designGG

### See Also

See Also `designScore`

---

[Package *designGG* version 1.0-02 Index]
