# Supplementary material for: designGG: an R-package and web tool for the optimal design of genetical genomics experiments
Source: BMC Bioinformatics. 2009 Jun 18;10:188. doi: 10.1186/1471-2105-10-188 (PMC2706229; doi:10.1186/1471-2105-10-188)
Supplement: Additional file 1 — designGG: an R-package for the optimal design of genetical genomics experiments. DesignGG aims at finding an optimal design of genetical genomics experiments which maximize the power and resolution of detecting genetic, environmental and interaction effects. This will help to achieve high power and more accurate estimates of the effects of interesting factors, and thus yield a more reliable biological interpretation of data. [file 1471-2105-10-188-S1.zip › designGG/html/plotAllScores.html]

R: Plot scores profiles

|  |  |
| --- | --- |
| plotAllScores {designGG} | R Documentation |

## Plot scores profiles

### Description

Plot all scores and the temperature at each iteration during the simulated annealing
process.

### Usage

```
 plotAllScores(plot.obj,fileName=NULL)
```

### Arguments

|  |  |
| --- | --- |
| `plot.obj` | a list containing: scores, cooling, startTemp, temperature, temperature.step, nIterations and optimality. Details can be found below. |
| `scores` | A- or D- optimality score of all accepted designs during optimization process. |
| `cooling` | describes the cooling step in the Simulated Annealing, defined as (new.score $-$ now.score)/ now.score. |
| `startTemp` | starting temperature of the simulated annealing process. |
| `temperature` | final temperature that the simulated annealing reaches. |
| `temperatureStep` | temperature decreasing step in the simulated annealing (SA) process. |
| `nIterations` | number of iterations in the simulated annealing method. |
| `optimality` | type of optimality, i.e. "A" (A-optimality) or "D" (D-optimality). A-optimality minimizes $Trace((X'X)^{-1})$, which corresponds to minimum average variance of the parameter estimates. D-optimality minimizes $det(X'X)^{-1}$, which corresponds to minimum generalized variance of the parameter estimates. |
| `fileName` | the final optimal design table(s) in `csv` format and a plot (in `png` format) of the all scores during SA process (if `plotScores` = T) will be produced. The users can specify the table and plot name by setting `fileName`. If `NULL` (default) it produces files starting with `"myDesignGG"`. |

### Value

Draw a plot that visualizeds the scores (y-axis) at each iteration during
the simulated annealing process (x-axis is time of moving)

### Note

The calculation of score is dependent on the choice of optimality.  
Cooling is defined as (newScore $-$ nowScore)/nowScore.

### Author(s)

Yang Li <yang.li@rug.nl>, Gonzalo Vera <gonzalo.vera.rodriguez@gmail.com>   
Rainer Breitling <r.breitling@rug.nl>, Ritsert Jansen <r.c.jansen@rug.nl>

### References

Y. Li, R. Breitling and R.C. Jansen. Generalizing genetical
genomics: the added value from environmental perturbation, Trends Genet
(2008) 24:518-524.   
Y. Li, M. Swertz, G. Vera, J. Fu, R. Breitling, and R.C. Jansen. designGG:
An R-package and Web tool for the optimal design of genetical genomics
experiments. (submitted)   
http://gbic.biol.rug.nl/designGG

---

[Package *designGG* version 1.0-02 Index]
