# Supplementary material for: designGG: an R-package and web tool for the optimal design of genetical genomics experiments
Source: BMC Bioinformatics. 2009 Jun 18;10:188. doi: 10.1186/1471-2105-10-188 (PMC2706229; doi:10.1186/1471-2105-10-188)
Supplement: Additional file 1 — designGG: an R-package for the optimal design of genetical genomics experiments. DesignGG aims at finding an optimal design of genetical genomics experiments which maximize the power and resolution of detecting genetic, environmental and interaction effects. This will help to achieve high power and more accurate estimates of the effects of interesting factors, and thus yield a more reliable biological interpretation of data. [file 1471-2105-10-188-S1.zip › designGG/html/temperatureStep.html]

R: Calculate the temperature decreasing step for simulated annealing process

|  |  |
| --- | --- |
| temperatureStep {designGG} | R Documentation |

## Calculate the temperature decreasing step for simulated annealing process

### Description

Calculate the temperature decreasing step for simulated annealing process.
This is a subfunction needed for `designGG`, but is not directly used.

### Usage

```
temperatureStep(startTemp, maxTempStep, endTemp, nIterations)
```

### Arguments

|  |  |
| --- | --- |
| `startTemp` | starting temperature of simulated annealing process. |
| `maxTempStep` | maximum temperature decreasing step for simulated annealing process. The parameter ensures that the multiplicative cooling factor is not smaller than this value. If nIterations is too small, the preferred final temperature (endTemp) may not be reached. See Wit and McClure (2004) for details. |
| `endTemp` | ending temperature of simulated annealing process. An important optimization parameter. Setting this parameter closer to zero. See Wit and McClure (2004) for details |
| `nIterations` | number of iterations in the simulated annealing method. |

### Value

A temperature decreasing step in the simulated annealing process.

### Author(s)

Yang Li <yang.li@rug.nl>, Gonzalo Vera <gonzalo.vera.rodriguez@gmail.com>   
Rainer Breitling <r.breitling@rug.nl>, Ritsert Jansen <r.c.jansen@rug.nl>

### References

Y. Li, M. Swertz, G. Vera, J. Fu, R. Breitling, and R.C. Jansen. designGG:
An R-package and Web tool for the optimal design of genetical genomics
experiments. (submitted)   
http://gbic.biol.rug.nl/designGG   
Y. Li, R. Breitling and R.C. Jansen. Generalizing genetical
genomics: the added value from environmental perturbation, Trends Genet
(2008) 24:518-524.   
E. Wit and J. McClure. Statistics for Microarrays: Design, Analysis
and Inference. (2004) Chichester: Wiley.

### See Also

`designGG`

---

[Package *designGG* version 1.0-02 Index]
