# Supplementary material for: designGG: an R-package and web tool for the optimal design of genetical genomics experiments
Source: BMC Bioinformatics. 2009 Jun 18;10:188. doi: 10.1186/1471-2105-10-188 (PMC2706229; doi:10.1186/1471-2105-10-188)
Supplement: Additional file 1 — designGG: an R-package for the optimal design of genetical genomics experiments. DesignGG aims at finding an optimal design of genetical genomics experiments which maximize the power and resolution of detecting genetic, environmental and interaction effects. This will help to achieve high power and more accurate estimates of the effects of interesting factors, and thus yield a more reliable biological interpretation of data. [file 1471-2105-10-188-S1.zip › designGG/html/updateDesign.html]

R: Updates current design

|  |  |
| --- | --- |
| updateDesign {designGG} | R Documentation |

## Updates current design

### Description

Updates current experimental design (including `array.allocation` and `condition.allocation`).

### Usage

```
  updateDesign( array.allocation, condition.allocation, nRILs, 
                nSlides, nEnvFactors, nTuple, btwoColorArray )
```

### Arguments

|  |  |
| --- | --- |
| `array.allocation` | matrix with nArray rows and nRIL columns. Elements of 1/0 indicate this RIL (or strain) is/not selected for this array. |
| `condition.allocation` | matrix with nCondition rows and nRIL columns. Elements of 1/0 indicate this RIL (or strain) is/not selected for this condition. |
| `nRILs` | number of RILs (or strains) available for the experiment. |
| `nSlides` | total number of slides available for experiment. |
| `nEnvFactors` | number of environmental factors, an integer bewteen 1 and 3. When `nEnvFactors` is 1 and the number of levels for the enviromental factor (`nLevels`)is 1, there is one condition in the experiment (i.e. no enviromental perturbation) and thus only genetic factor will be considered in the algorithm. When `nEnvFactors` is 1 and nLevels is larger than 1 or `nEnvFactors` is larger than 1, all main factor(s) and interacting facotr(s) will be included. Examples: If there is a temperature perturbation, then `nEnvFactors` is 1; If there is both temperature and drug treatment perturbation, then `nEnvFactors` is 2. |
| `nTuple` | average number of RILs (or strains) to be assigned onto each condition.   `nTuple` should be a real number which is larger than 1.  If `nTuple` < 1, the algorithm will stop and show the message,   `warning: "The number of slides is too small to perform the experiment."` |
| `bTwoColorArray` | binary variable indicating experiment type:   `bTwoColorArray <- T` #for dual channel experiment   `bTwoColorArray <- F` #for single channel experiment |

### Details

This function calls two subfunctions: `conditionUpdate` and `arrayUpdate`.

### Value

a list with two elements, `array.allocation` and `condition.allocation`.

### Author(s)

Yang Li <yang.li@rug.nl>, Gonzalo Vera <gonzalo.vera.rodriguez@gmail.com>   
Rainer Breitling <r.breitling@rug.nl>, Ritsert Jansen <r.c.jansen@rug.nl>

### References

Y. Li, R. Breitling and R.C. Jansen. Generalizing genetical
genomics: the added value from environmental perturbation, Trends Genet
(2008) 24:518-524.   
Y. Li, M. Swertz, G. Vera, J. Fu, R. Breitling, and R.C. Jansen. designGG:
An R-package and Web tool for the optimal design of genetical genomics
experiments. (submitted)   
http://gbic.biol.rug.nl/designGG

---

[Package *designGG* version 1.0-02 Index]
