# Supplementary material for: designGG: an R-package and web tool for the optimal design of genetical genomics experiments
Source: BMC Bioinformatics. 2009 Jun 18;10:188. doi: 10.1186/1471-2105-10-188 (PMC2706229; doi:10.1186/1471-2105-10-188)
Supplement: Additional file 1 — designGG: an R-package for the optimal design of genetical genomics experiments. DesignGG aims at finding an optimal design of genetical genomics experiments which maximize the power and resolution of detecting genetic, environmental and interaction effects. This will help to achieve high power and more accurate estimates of the effects of interesting factors, and thus yield a more reliable biological interpretation of data. [file 1471-2105-10-188-S1.zip › designGG/html/variableNames.html]

R: Generate variable names for all factors

|  |  |
| --- | --- |
| variableNames {designGG} | R Documentation |

## Generate variable names for all factors

### Description

Generate variable names for genetic, environmental factors and interacting
terms.

### Usage

```
variableNames(nEnvFactors)
```

### Arguments

|  |  |
| --- | --- |
| `nEnvFactors` | number of environmental factors, an integer bewteen 1 and 3. When `nEnvFactors` is 1 and the number of levels for the enviromental factor (`nLevels`)is 1, there is one condition in the experiment (i.e. no enviromental perturbation) and thus only genetic factor will be considered in the algorithm. When `nEnvFactors` is 1 and nLevels is larger than 1 or `nEnvFactors` is larger than 1, all main factor(s) and interacting facotr(s) will be included. Examples: If there is a temperature perturbation, then `nEnvFactors` is 1; If there is both temperature and drug treatment perturbation, then `nEnvFactors` is 2. |

### Details

generates names for variables, a vector with the length of (variableNumber+1).

### Value

When `nEnvFactors` = 1 and `nLevels` = 1, there is no environmetal pertubation in the experimental.
Then we re-define `nEnvFactors` to be 0 within the algorithm. Accordingly, `variableNumber` = 1, and
`variableNames` is one genetic factor "Q".   
When `nEnvFactors` = 1, `variableNumber` = 3, and
`variableNames` are one genetic factor "Q", one environmental factor "F",
and one interacting factor "QxF".   
When `nEnvFactors` = 2, `variableNumber` = 7, and `variableNames` are one genetic factor "Q",
two environmental factors "F1" and "F2",
three two-way interacting factors "QF1", "QF2", "F1F2",
and one three way interacting factors "QxF1xF2".   
When `nEnvFactors` = 3, `variableNumber` = 15, and
`variableNames` are one genetic factor "Q",
three environmental factors "F1", "F2" and "F3",
six two-way interacting factors "QF1", "QF2", "QF3", "F1F2",
"F2F3" and "F1F3",
four three-way interacting factors "QxF1xF2", "QxF1xF3",
"QxF2xF3", "F1xF2xF3"
and one four-way interacting factors "QxF1xF2xF3".

### Author(s)

Yang Li <yang.li@rug.nl>, Gonzalo Vera <gonzalo.vera.rodriguez@gmail.com>   
Rainer Breitling <r.breitling@rug.nl>, Ritsert Jansen <r.c.jansen@rug.nl>

### References

Y. Li, R. Breitling and R.C. Jansen. Generalizing genetical
genomics: the added value from environmental perturbation, Trends Genet
(2008) 24:518-524.   
Y. Li, M. Swertz, G. Vera, J. Fu, R. Breitling, and R.C. Jansen. designGG:
An R-package and Web tool for the optimal design of genetical genomics
experiments. (submitted)   
http://gbic.biol.rug.nl/designGG

### See Also

`variableNumber`

---

[Package *designGG* version 1.0-02 Index]
