# Supplementary material for: designGG: an R-package and web tool for the optimal design of genetical genomics experiments
Source: BMC Bioinformatics. 2009 Jun 18;10:188. doi: 10.1186/1471-2105-10-188 (PMC2706229; doi:10.1186/1471-2105-10-188)
Supplement: Additional file 1 — designGG: an R-package for the optimal design of genetical genomics experiments. DesignGG aims at finding an optimal design of genetical genomics experiments which maximize the power and resolution of detecting genetic, environmental and interaction effects. This will help to achieve high power and more accurate estimates of the effects of interesting factors, and thus yield a more reliable biological interpretation of data. [file 1471-2105-10-188-S1.zip › designGG/html/variableNumber.html]

R: Compute the number of variables in the experiment

|  |  |
| --- | --- |
| variableNumber {designGG} | R Documentation |

## Compute the number of variables in the experiment

### Description

When `nEnvFactors` = 1 and `nLevels` = 1, there is no environmetal
pertubation in the experimental.
Then we re-define `nEnvFactors` to be 0 within the algorithm.
`nEnvFactors` = 0, only genetic factor is considered.   
`nEnvFactors` > 1, genetic and environmental facotrs, and all possible
interacting factors are considered.

### Usage

```
  variableNumber( nEnvFactors )
```

### Arguments

|  |  |
| --- | --- |
| `nEnvFactors` | number of environmental factors, an integer.  When nEnvFactors is between 0 and 3, all main factors and interacting factors will be included. |

### Value

`nEnvFactors` = 1, `variableNumber` = 3 (one genetic factor Q, one environmental
factor F, and one interacting factor QxF)  
`nEnvFactors` = 2, `variableNumber` = 7   
`nEnvFactors` = 3, `variableNumber` = 15

### Author(s)

Yang Li <yang.li@rug.nl>, Gonzalo Vera <gonzalo.vera.rodriguez@gmail.com>   
Rainer Breitling <r.breitling@rug.nl>, Ritsert Jansen <r.c.jansen@rug.nl>

### References

Y. Li, R. Breitling and R.C. Jansen. Generalizing genetical
genomics: the added value from environmental perturbation, Trends Genet
(2008) 24:518-524.   
Y. Li, M. Swertz, G. Vera, J. Fu, R. Breitling, and R.C. Jansen. designGG:
An R-package and Web tool for the optimal design of genetical genomics
experiments. (submitted)   
http://gbic.biol.rug.nl/designGG

### See Also

`variableNames`

---

[Package *designGG* version 1.0-02 Index]
